# Declining incidence of imported malaria in the Netherlands, 2000-2007

**DOI:** 10.1186/1475-2875-9-300

**Published:** 2010-10-28

**Authors:** Gini GC van Rijckevorsel, Gerard JB Sonder, Ronald B Geskus, Jose CFM Wetsteyn, Robert J Ligthelm, Leo G Visser, Monique Keuter, Perry JJ van Genderen, Anneke van den Hoek

**Affiliations:** 1 Public Health Service Amsterdam, Department of Infectious Diseases, Amsterdam, The Netherlands; 2 Malaria Working Group of National Coordination Centre for Travellers' Health Advice (LCR), Amsterdam, The Netherlands; 3 Academic Medical Center, Department of Internal Medicine, Division of Infectious Diseases, Tropical Medicine and AIDS, Amsterdam, The Netherlands; 4 Academic Medical Center, Department of Clinical Epidemiology, Biostatistics and Bioinformatics, Amsterdam, The Netherlands; 5 Tropvacc BV, Rotterdam, The Netherlands; 6 Leiden University Medical Centre, Department of Infectious Disease, Section Travel Medicine, The Netherlands; 7 Radboud University Nijmegen Medical Center, Department of Medicine, Division of General Internal Medicine, Nijmegen; 8 Department of Internal Medicine, Harbour Hospital and Institute for Tropical Diseases, Rotterdam, The Netherlands

## Abstract

**Background:**

To describe the epidemiology and trends of imported malaria in the Netherlands from 2000 through 2007.

**Methods:**

Based on national surveillance data regarding all reported infections of imported malaria, diagnosed 2000 through 2007, incidence and trends of imported malaria in the Netherlands were estimated. Travellers statistics were used to estimate incidence, and data on malaria chemoprophylaxis prescriptions were used to estimate the number of unprotected travellers.

**Results:**

Importation of malaria to the Netherlands is declining even as more travellers visit malaria-endemic countries. On average, 82% were acquired in sub-Saharan Africa, and 75% were caused by *Plasmodium falciparum*. The overall incidence in imported falciparum malaria fell from 21.5 to 6.6/10,000 of unprotected travellers. The percentage of unprotected travellers rose from 47% to 52% of all travellers. The incidence of imported falciparum infections is greatest from Middle and West Africa, and decreased from 121.3 to 36.5/10,000 travellers. The import of malaria from this region by immigrants visiting friends and relatives (VFR) decreased from 138 infections in 2000, to 69 infections in 2007.

**Conclusion:**

The annual number of imported malaria shows a continuing declining trend, even with an increasing number of travellers visiting malaria endemic countries. VFR import less malaria than previously, and contribute largely to the declining incidence seen. The decline is not readily explained by increased use of chemoprophylaxis and may reflect a reduced risk of infection due to decreasing local malaria transmission as observed in some malaria endemic areas. Nevertheless, the increasing number of unprotected travellers remains worrisome.

## Background

Malaria is the world's most important parasitic disease and is endemic in more than 105 countries. About 3.3 billion people, or half of the world's population, are at risk of malaria. Every year there are about 250 million malaria cases and nearly one million deaths [1,2], mostly in malaria-endemic regions. An infection acquired in an endemic region, but diagnosed in a non-endemic country, usually after development of clinical symptoms, is defined as imported malaria [1,3,4]. Such diagnoses may occur either in travellers who have recently toured or visited endemic regions or in travellers from endemic regions who are visiting a non-endemic country. Travellers from non-endemic regions are generally advised to use personal protective measures and malaria chemoprophylaxis when visiting malaria-endemic regions. Yet despite the availability of adequate chemoprophylactic drugs in the Netherlands and other non-endemic countries, imported malaria continues to cause considerable morbidity and mortality among returning travellers. Immigrants from endemic countries and returning to their country of origin to visit friends and relatives (VFR) are a particular risk group among these [5,6]. From the 1970s onward, the annual importation of malaria to Europe showed a steady increase. In the late 1990s, these imported cases in Europe were estimated to number at least 10,000 to 13,000 per year, excluding many that are believed to be unreported [7-9].

The aim of this research is to present a comprehensive overview of trends in imported malaria in the Netherlands in the period from 2000 through 2007. In addition, the annual incidence of *Plasmodium falciparum *infections is estimated using travel statistics regarding the annual number of travellers to malaria-endemic regions, as well as data from Dutch pharmacies regarding annual prescriptions for malaria chemoprophylaxis.

## Methods

### Malaria infections

In the Netherlands, all laboratory-confirmed infections of malaria are reported to the National Institute for Public Health and the Environment (RIVM). Since such reports became compulsory in 1932, the system of reporting has changed over the years. In order to minimize underreporting of malaria infections in the Netherlands, the reporting system was changed in 1999 from doctor-based to laboratory-based reporting. Underreporting before that time was estimated to be about 60% [10]. All infections reported between January 1, 2000, and December 31, 2007 were analysed, using the following surveillance data: date of laboratory confirmation of the *Plasmodium *species (results of conventional microscopy with subsequent specification of the *Plasmodium *species), patient data (gender, date of birth, country of birth, and country of mother's birth), and travel information (most likely country of infection, reason for travel). Infected persons were classified as to ethnic origin based on the reported country of their birth and/or their mother's birth. They were classified into four categories based on their reason for travel; Western tourists visiting endemic countries; Dutch expatriates living in endemic countries (including military and airline personnel); visiting residents from endemic countries (including new immigrants, asylum seekers, refugees); and immigrants (or their descendants) from endemic countries to the Netherland who visit friends and relatives (VFR) in those countries. In this report, these categories will be referred to as, tourists, expatriates, visitors and VFR, respectively. In the period 2000-2003, data on some of these variables were missing. In 2003, a new digital reporting system was introduced, providing more complete information.

### Traveller statistics

Only data on malaria-endemic countries, for which the Malaria Working Group of Dutch the National Coordination Centre for Travellers' Health Advice (LCR) advises travellers to take malaria chemoprophylaxis were used[11]. See additional file 1 for a list of all travel destinations included. When no country-specific travel data were available, regional data were used instead. Countries were grouped into continental regions and sub-regions according to the composition of macro-geographical regions described by the United Nations Statistics Division [12].

### Malaria chemoprophylaxis prescriptions

Data on the number of annual prescriptions for malaria chemoprophylaxis (atovaquone/proguanil, mefloquine, and proguanil), collected from pharmacies in the Netherlands by the Foundation for Pharmaceutical Statistics (SFK) [13], were employed to estimate the annual number of travellers using the chemoprophylaxis. Chloroquine was not included because it is generally prescribed in combination with proguanil, and not as a single chemoprophylactic drug. Doxycycline was not included, because it is not recommended as a first drug of choice, and according to National Coordination Centre for Travellers' Health Advice (LCR), only sporadically prescribed as a malaria chemoprophylactic drug. It is mostly prescribed for other indications than malaria prophylaxis.

### Data analysis

The incidence of imported *P. falciparum *malaria in the Netherlands was estimated using the annual number of reported *P. falciparum *infections as numerator and the number of travellers to malaria-endemic regions as denominator. Imported infections in visiting residents from malaria-endemic countries were excluded from this part of the analysis, as visiting residents from malaria-endemic countries are no part of the travellers statistics.

Incidence specific to a region or a sub-region was estimated using the number of reported *P. falciparum *infections from that region or sub-region as numerator and the annual number of travellers to that region or sub-region as denominator.

In addition, assuming that *P. falciparum *infection mainly occurs in travellers not using chemoprophylaxis, the incidence of imported *P. falciparum *malaria in unprotected travellers from the Netherlands was calculated. The annual number of unprotected travellers was estimated by deducting the number of annual prescriptions for malaria chemoprophylaxis from the total of travellers to malaria-endemic regions., A region-specific incidence for "unprotected" travellers only could not be calculated, because data on prescriptions from Dutch pharmacies do not include data on destination. Trends in infection probability (incidence) were modelled via Poisson regression. Trends in relative contribution of reason for travel were modelled via multinomial logistic regression. All time trends were fitted using restricted cubic splines, to allow for smoothly varying trends over time [14]. Analyses were performed in the R statistical package.

## Results

### Imported infections of malaria 2000-2007

From 2000 through 2007, a total of 2,847 persons were diagnosed with malaria in the Netherlands. The annual number of imported malaria infections fell from 535 in 2000 to 197 in 2007. Most infections (92% or 2,624) occurred in adults; 5% occurred in children aged 6-15 years, and 3% in children five years or younger. The median age of infection was 36 years, and 33% of patients were female. Table 1 shows the annual number of reported infections by *Plasmodium *species. As in 11% (322) of all confirmed infections the causative species was not specified, mainly in the years 2000 through 2003, estimates were calculated. In the column 'adjusted' of Table 1, the unknown *Plasmodium *species were proportionally distributed among the four species, based on their respective annual contribution. Most recorded infections (2,131 or 75%) were caused by *P. falciparum*. The remainder was caused by *Plasmodium vivax *(15%), *Plasmodium ovale *(7%), and *Plasmodium malariae *(3%). A very small proportion of all infections (20 or 0.7%) was attributable to mixtures of species, most of which involved *P. falciparum*. Despite a decrease in the absolute number of infected patients, the relative distribution of the causative species remains fairly stable over the time period studied (p = 0.58).

**Table 1:** Reported infections of malaria in the Netherlands. 2000-2007 In the column 'adjusted' unknown *Plasmodium *species were proportionally distributed among the four species based on their respective annual contribution.

Species	*P. falciparum*		*P. ovale*		*P. vivax*		*P. malariae*		Total	Not specified
Year	No.	No. corrected (%)	No.	No. corrected (%)	No.	No. corrected (%)	No.	No. corrected (%)		
**2000**	295	413 (77,2%)	28	39 (7,3%)	52	73 (13,6%)	7	11 (1,8%)	535	(153)
**2001**	313	390 (71,3%)	34	42 (7,7%)	78	97 (17,8%)	14	17 (3,2%)	547	(108)
**2002**	269	294 (74,3%)	29	32 (8,0%)	53	58 (14,6%)	11	12 (3,0%)	396	(34)
**2003**	244	255 (75,1%)	24	25 (7,4%)	49	51 (15,1%)	8	8 (2,5%)	340	(15)
**2004**	199	201 (68,6%)	25	25 (8,6%)	60	61 (20,7%)	6	6 (2,1%)	293	(3)
**2005**	232	235 (78,9%)	22	22 (7,5%)	33	33 (11,2%)	7	7 (2,4%)	298	(4)
**2006**	186	189 (78,5%)	14	14 (5,9%)	30	31 (12,7%)	7	7 (3.0%)	241	(4)
**2007**	151	152 (77,0%)	8	8 (4,1%)	33	33 (16,8%)	4	4 (2,0%)	197	(1)
**Total**	**1,889**	**2,130 (74,8%)**	**184**	**208 (7,3%)**	**388**	**437 (15,4%)**	**64**	**72 (2,5%)**	**2,847**	**(322)**

### Region of infection

In 88% (2,511) of the records, the most likely country of infection was supplied. Table 2 shows the continental region and sub-region where the infections were acquired, by *Plasmodium *species. Most infections were acquired in Africa, (2,068 or 82%), followed by Asia (298 or 12%) and Middle and South America (142 or 6%). From 2000 through 2007, the annual number of imported *Plasmodium *infections from Africa almost halved, from 310 to 159 infections per year. Infections from Middle and South America fell from 27 in 2000 to seven in 2007. Imported infections from Asia varied, ranging from 18 to 63 infections per year.

**Table 2:** Reported imported infections of malaria by continent and sub-region of infection, and by species, The Netherlands, 2000-2007.

Region	*P. falciparum*	*P. ovale*	*P. vivax*	*P. malariae*	Species unknown	Total
**Africa**	1,658 (94%)	157 (94%)	78 (22%)	47 (78%)	128 (80%)	2,068 (82%)
*Middle & West Africa*^1^	*1413*	*110*	*23*	*39*	*89*	*1674*
*East & Southern Africa*^2^	*245*	*47*	*55*	*8*	*39*	*394*
**America**	43 (2%)	2 (1%)	79 (23%)	7 (12%)	11 (7%)	142 (6%)
*Middle America*^3^	*4*	*0*	*17*	*0*	*5*	*24*
*South America*^4^	*39*	*2*	*62*	*7*	*6*	*118*
**Asia**	71 (4%)	9 (5%)	192 (55%)	5 (8%)	21 (13%)	298 (12%)
*South Asia*^5^	*20*	*0*	*57*	*3*	*9*	*89*
*South East Asia*^6^	*51*	*9*	*135*	*2*	*12*	*209*
**Oceania**	2 (0.1%)	0 (0.0%)	0 (0.0%)	1 (2%)	0 (0.0%)	3 (0.1%)
**Sub total**	**1,774 (100%)**	**169 (100%)**	**349 (100%)**	**60 (100%)**	**160 (100%)**	**2,511 (100%)**
Region of infection unknown	115	15	39	4	162	336
**Total**	**1,889**	**184**	**388**	**64**	**322**	**2,847**

^1^Angola, Benin, Burkina Faso, Cameroon, Cape Verde, Central African Republic, Chad, Côte d'Ivoire, Congo, the Democratic republic of Congo, Gabon, The Gambia, Ghana, Equatorial Guinea, Guinea, Guinea-Bissau,, Liberia, Mali, Mauritania, Niger, Nigeria, São Tomé and Principe, Senegal, Sierra Leone, Togo.

^2^Botswana, Burundi, Djibouti, Eritrea, Ethiopia, Kenya, Lesotho, Malawi, Mozambique, Nauru, Namibia, Rwanda, South Africa, Somalia, Sudan, Swaziland, Tanzania, Uganda, Zimbabwe.

^3^Anguilla (U.K.), Antigua and Barbuda, Aruba, the Bahamas, Barbados, Belize, Bermuda (U.K.), Cayman Islands (U.K.), Costa Rica, Cuba, Dominica, Dominican Republic, El Salvador, Grenada, Guadeloupe, Guatemala, Haiti, Honduras, Jamaica, Martinique (France), Mexico, Montserrat (U.K.), Netherlands Antilles, Nicaragua, Panama, Puerto Rico (U.S.), Saint Kitts and Nevis, Saint Lucia, Saint Vincent and the Grenadines, Trinidad and Tobago, Turks and Caicos Islands (U.K.), Virgin Islands, British, Virgin Islands, U.S.

^4^Argentina, Bolivia, Brazil, Chile, Colombia, Easter Island (Chile), Ecuador, Falkland Islands (U.K.), French Guiana (France), Guyana, Paraguay, Peru, South Sandwich Islands, Suriname, Uruguay, Venezuela.

^5^Afghanistan, Bangladesh, Bhutan, India, Iraq, Iran, Maldives, Nepal, Oman, Pakistan, Sri Lanka.

^6^Brunei, Burma (Myanmar), Cambodia, Indonesia, Laos, Malaysia, Philippines, Singapore, Thailand, Timor-Leste (East Timor), Vietnam.

Infections acquired in Asia were for the greater part (64%) caused by *P. vivax*, whereas those acquired in Africa were for the greater part (80%) caused by *P. falciparum. *Almost all *P. falciparum *infections (1,658 or 94%) were acquired in Africa. Of these, 80% (1,337) were acquired in the West African sub-region, with most coming from Ghana (550) followed by Nigeria (280) and the Gambia (126). Importation of *P. falciparum *from West Africa decreased from 182 infections in 2000 to 124 infections in 2007. The decrease was seen for all countries in this sub-region except Nigeria, which showed an increase.

### Reason for travel

Information regarding the reason of the travel was available for 82% (2,346) of cases.

Of these, 53% were adults and children who had emigrated from malaria-endemic countries, settled in the Netherlands, and were subsequently visiting friends and relatives. Infections in these VFR declined from 210 in 2000 to 77 in 2007. Among Western tourist travellers (666 or 28%), infections declined from 122 to 53 over the study period. In expatriates (309 or 13%), infections remained stable, ranging from 32 and 50 per year.

**Figure 1:**
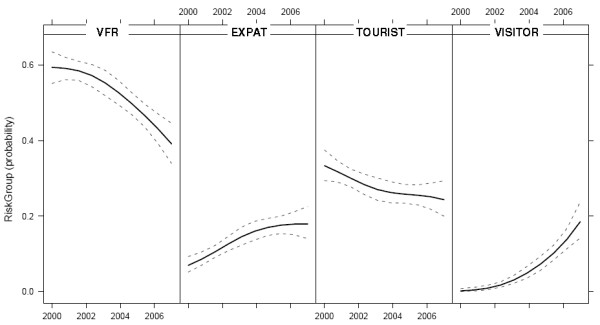
**Fitted trends in probability of infection for the four categories of patients with imported malaria, the Netherlands, 2000-2007. **The probability of infection is shown with 95% confidence intervals.

In 119 cases (5%), the diagnosis of malaria was made in visiting residents from malaria endemic countries, mostly from the African region. The number of infections diagnosed in visiting residents increased from three in 2000 to 24 in 2007. The relative contributions for all four categories of patients to the probability of an imported malaria infection were estimated. Figure 1 shows the fitted trends of the probability of infection for all four categories of patients with 95% confidence intervals. The probability of infection in VFR and Western tourists show a declining trend, whereas the probability of infection in expatriates and visiting residents from malaria endemic countries is increasing.

### Ethnic origin

Information on the ethnic origin of the patients was available in 75% (2,141) of the records. Of these patients, almost half (1,042 or 49%) were persons of Middle and West African origin, mainly from Ghana (452) and Nigeria (221), of which 98% and 95% acquired the infection in the country of origin, respectively. Of these, 11% (115/1,042) were aged 16 years or younger. The number of infections in persons of Middle and West African origin declined from 150 in 2000 to 80 in 2007, and in persons of East and Southern African origin from 22 in 2000 to 10 in 2007. Approximately a third (769 or 36%) of the infections were in persons of Dutch ethnic origin, of which 5% were in children aged 16 years or younger. The countries in which Dutch persons most frequently acquired infection were Ghana (102), Indonesia (78), or the Gambia (72). The number of infections in persons of Dutch ethnic origin fluctuated between 68 and 129. Few infections occurred in persons of South or South East Asian origin (83 or 4%; mainly from Afghanistan (14), India (18), and Indonesia (19)), and in persons of Middle or South American origin (58 or 3%; mainly Surinam (42)). Infections in all these groups decreased.

### Number of travellers and the use of malaria chemoprophylaxis

The number of travellers visiting the malaria-endemic regions for which the LCR recommends malaria chemoprophylaxis increased from 247,000 in 2000 to 384,000 in 2007 [12]. The number of travellers to the Middle and West African sub-region almost doubled over time from 16,000 to 31,000, but the proportion of travel to this region is rather small (range 3-13%) compared to other the regions.

**Figure 2:**
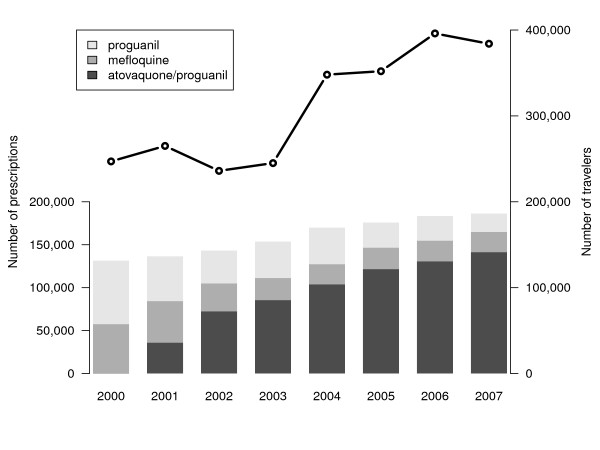
**Number of collected prescriptions for malaria chemoprophylaxis (shown in bars), and total number of travellers to malaria-endemic regions (shown in line), the Netherlands, 2000-2007**.

In the same period, the number of prescriptions for malaria chemoprophylaxis issued by Dutch pharmacies rose steadily by 42%, from 131,400 in 2000 to 186,300 in 2007. In 2000, the combination drug atovaquone/proguanil was registered for the use of malaria chemoprophylaxis, whereupon it gradually replaced the number of prescriptions for mefloquine and proguanil. In 2007, 76% of all prescriptions were atovaquone/proguanil. Figure 2 shows the proportional change in the use of malaria chemoprophylaxis and the absolute growth of the number of travellers to malaria-endemic countries. Despite the increased number of prescriptions for malaria chemoprophylaxis, the proportion of protected travellers decreased from 53% (115,600) of all travellers in 2000 to 48% (197,700) of all travellers in 2007.

### Trends in incidence of imported *Plasmodium falciparum *malaria

**Figure 3:**
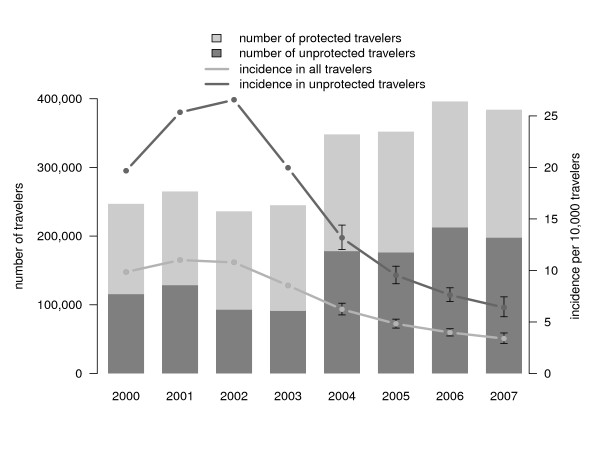
Overall incidence of imported *Plasmodium falciparum *malaria in the Netherlands, 2000-2007, and number of travellers to malaria endemic regions.

**Figure 4:**
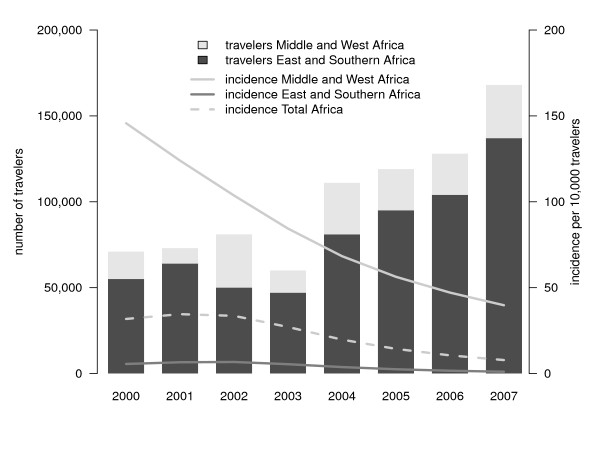
**Region-specific of imported *Plasmodium falciparum *malaria in the Netherlands, 2000-2007, and number of travellers to malaria endemic regions - Africa**.

**Figure 5:**
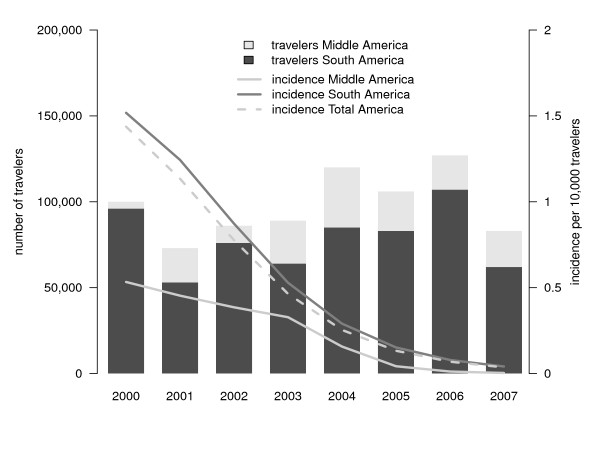
**Region-specific of imported *Plasmodium falciparum *malaria in the Netherlands, 2000-2007, and number of travellers to malaria endemic regions - Central and South America**.

**Figure 6:**
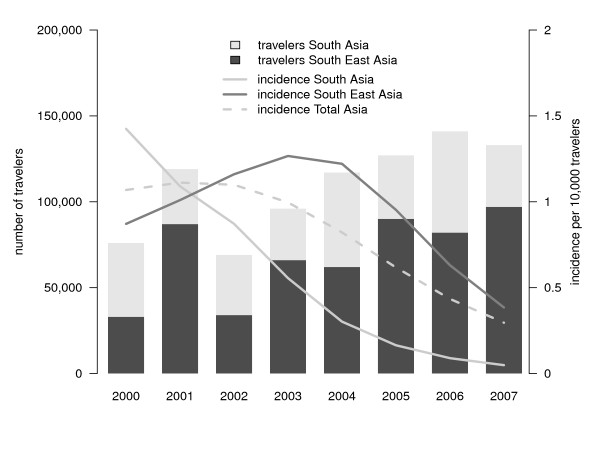
**Region-specific of imported *Plasmodium falciparum *malaria in the Netherlands, 2000-2007, and number of travellers to malaria endemic regions - Asia**.

Figure 3 shows the estimated annual incidence of *P. falciparum *malaria imported in unprotected travellers and in all travellers from the Netherlands, combined with the number of travellers. The estimated incidence was highest in 2002 (27.5/10,000 unprotected travellers) but dropped from 21.5 in 2000 to 6.6 infections per 10,000 unprotected travellers in 2007 (p < 0.001). The incidence in all travellers (i.e., protected and unprotected) declined from 10.0 to 3.4 infections per 10,000 travellers (p < 0.001). This drop is for the most part caused by the decreased importation from Africa, in particular from Middle and West Africa (from 121.3/10,000 travellers in 2000 to 36.5/10,000 travellers in 2007, p < 0.001). Figures 4, 5 and 6 show the incidence changes per region and sub-region. Except for Asia (p = 0.15) and Middle America (p = 0.5), all areas showed significant declining trends (p < 0.001, except South East Asia, p = 0.03), however the number of infections were small for these regions.

## Discussion

The results of this study show a steep and declining trend in malaria infections since 2000. This is in striking contrast to the steady increase in imported cases that began in the 1970s [15]. Despite increasing travel to malaria-endemic countries, the estimated incidence of imported *P. falciparum *infections per 10,000 travellers declined from 10.0 in 2000 to 3.4 in 2007. This decrease is not readily explained by an increased uptake of malaria chemoprophylaxis, because the observed increase of prescriptions for chemoprophylaxis did not match the growth in travel. Moreover, despite the additional number of travellers at risk, the estimated incidence of imported *P. falciparum *fell from 21.5 to 6.6 infections per 10,000 unprotected travellers.

In this study, incidence estimates were calculated assuming that *P. falciparum *infection occurs only in travellers not using malaria chemoprophylaxis. Other studies confirm this [16-19], and several surveys show that up to 60% of travellers are not protected against malaria [20,21]. Of course it is likely that some of the imported infections in the Netherlands were caused by incorrect use of malaria chemoprophylaxis, rather than non-use. Improved drug compliance over time may have contributed to the observed decrease of imported malaria. It is likely that the increased use of atovaquone/proguanil, since 2000, has enhanced compliance with chemoprophylaxis [22,23], and in this way contributed to the decreased importation of malaria. Yet, some studies found no difference in compliance among the various prophylactic regimens [24-26]. Furthermore, how patients comply depends on many more variables than the chemoprophylactic drug itself, such as personal characteristics, perception of risk, and travel destination [27-29]. The risk of infection in travellers *not *using malaria chemoprophylaxis depends on other factors too. For example, the use of personal protective measures, such as impregnated bed nets and mosquito repellents, reduces the risk of infection considerably. Lastly, the risk of infection depends on the malaria endemicity, which varies per country, within a country or region, but also may depend on the season of travel. In some travel destination personal protective measures offer sufficient protection against infection. In conclusion, this may have led to an underestimation of the true incidence, but still allowed us to analyse the declining trends.

The drop in incidence of imported *P. falciparum *infections is greatest in travellers returning from Middle and West Africa, even though travel to this sub-region has doubled. Other studies likewise describe a declining incidence of malaria imported from West Africa [4,18,30]. Most malaria infections in the Netherlands are acquired in that region, and occurred in immigrants, mainly VFR from Ghana and Nigeria. The importation of malaria by VFR from this region decreased from 138 infections in 2000 to 69 infections in 2007, making a large contribution to the observed decline in incidence. Also among tourist travellers, the number of infections from this region (mainly from Ghana and the Gambia) decreased from 65 infections in 2000 to 25 infections in 2007.

An explanation may be a decreased risk of infection in their destination country [30]. Indeed, in some African countries a reduction in local malaria transmission is reported, especially in urban areas [31]. Some countries have achieved a high coverage of measures to control malaria, including extended use of bed nets treated with insecticide and improved access to malaria treatment, resulting in a large fall in the number of malaria cases and deaths between 2000 and 2007 [2,32]. At the same time in some other countries like Ghana and Nigeria, there was no evidence of a true reduction in malaria infections, and most parts of Africa are still considered areas of high endemicity [2,33]. Yet VFR may have improved their accommodations when visiting, e.g. by investing in better local housing, often in cities and urban areas, and having better access to protective measures like indoor spraying and insecticide-treated bed nets. In 2001, the public health service of Amsterdam studied factors determining the use of pre-travel preventive services by VFR traveling to West Africa from the Netherlands. The researchers concluded that some West Africans (in particular, non-Ghanaians, illegal immigrants, and immigrants leaving at short notice) were not consulting pre-travel preventive health services [34]. An explanation for the observed decrease in malaria imported by Ghanaian VFR may be that their uptake of malaria chemoprophylaxis has improved even more than in other groups.

A limitation of this study may be that the travellers statistics, based on the Continuous Holiday Survey, did not include non-Dutch and illegal immigrants. In the Netherlands, a large proportion (50%) of the resident West Africans have no legal status, although this percentage is less among people from Ghana than those from Nigeria [34]. (Illegal) immigrants were classified in the VFR category, but were not represented in the denominator. Any resulting overestimation of the incidence of malaria in West African travellers would be small, as illegal immigrants lack official documents permitting regular travel.

Another limitation of this study is that for some continental regions, country-specific travellers statistics were unavailable. In estimating the incidence specific to regions and sub-regions, only travellers statistics of countries for which the Malaria Working Group of the Dutch LCR advises malaria chemoprophylaxis were used. This produced however some denominator problems. For South America data for the whole sub-region were used as travel data to malaria-endemic and non-endemic countries could not be separated,. As mentioned previously this may have led to an underestimation of the true incidence of infection from the individual malaria-endemic countries.

## Conclusion

The declining incidence of imported malaria is not easily explained and likely to be multi-factorial. The decline is positive, but the increasing number of travellers from the Netherlands using no malaria chemoprophylaxis remains a concern. Despite the current optimism about decreasing malaria transmission in several parts of the world, vigilance is needed for the growing lack of awareness among travellers and, therefore, the growing need for prophylactic measures. In 2008, a cluster of 56 European tourist travellers returned from Gambia with *P. falciparum*, of which three died. All patients did not use, or wrongly used chemoprophylaxis [35]. For some malarious destinations, such as the Indian subcontinent and Middle and South America, less strict guidelines on malaria prophylaxis seem plausible [36,37]. However, an improved effort is needed to increase awareness and protection among the growing number of travellers to Africa and other areas endemic for *P. falciparum*.

## Competing interests

The authors declare that they have no competing interests.

## Authors' contributions

GGCvR performed the data analysis and wrote the first draft of the manuscript. RBG contributed to the data analysis. GBS and AvdH made substantial changes to the manuscript. All members of the Malaria Working Group of the LCR: JCFMW, RJL, LGV, PJJvG, MK have contributed to the manuscript.

All authors, including the members of the Malaria Working Group of the LCR have seen and approved the final version.

## Supplementary Material

Additional file 1 **Travel destinations used for travellers statistics**. Click here for file
