# Supplementary material for: Declining incidence of imported malaria in the Netherlands, 2000-2007
Source: Malar J. 2010 Oct 28;9:300. doi: 10.1186/1475-2875-9-300 (PMC2988037; doi:10.1186/1475-2875-9-300)
Supplement: Additional file 1 — Travel destinations used for travellers statistics. [file 1475-2875-9-300-S1.PDF]

Additional file 1:

Travel destinations used for travellers statistics:

Africa : Angola, Benin, Botswana, Burkina Faso, Burundi, Cameroon, Cape Verde, Central African Republic, Chad, Côte d'Ivoire, Congo, the Democratic republic of Congo, Djibouti, Eritrea, Ethiopia Gabon, the Gambia, Equatorial Guinea, Guinea, Guinea-Bissau, Kenya, Lesotho Liberia, Malawi, Mali, Mauritania, Mozambique, Namibia, Niger, Nigeria, Rwanda, São Tomé and Príncipe, Senegal, Sierra Leone, South Africa, Somalia, Sudan, Swaziland, Tanzania, Togo, Uganda, Zimbabwe.

Asia: Afghanistan, Bangladesh, Bhutan, Brunei, Burma (Myanmar), Cambodia, India, Indonesia, Iraq, Iran, Laos, Maldives Nepal, Oman, Pakistan, Philippines, Singapore, Sri Lanka, Timor-Leste (East Timor), Vietnam.

Middle and South America : Argentina, Belize, Bolivia, Brazil, Chile, Colombia, Costa Rica, Ecuador, El Salvador, French Guiana (France), Guatemala, Guyana, Honduras, Nicaragua, Panama, Paraguay, Peru, Suriname, Uruguay, Venezuela.
